# Return-to-School Evaluation Criteria for Children With Suspected Coronavirus Disease 2019

**DOI:** 10.3389/fpubh.2020.618642

**Published:** 2021-01-08

**Authors:** Vasiliki Vlacha, Gavriela Maria Feketea

**Affiliations:** ^1^Department of Early Years Learning and Care, University of Ioannina, Ioannina, Greece; ^2^Ph.D School, “Iuliu Hatieganu” University of Medicine and Pharmacy, Cluj-Napoca, Romania; ^3^Paediatric Department, Karamandanio Children's Hospital of Patras, Patras, Greece

**Keywords:** COVID 19, school age children, return-to-school, SARS-CoV-2 RT-PCR, criteria

During the coming winter, the spread of Coronavirus disease 2019 (COVID-19) will co-exist with other common viral infections. Even if most of the children with COVID-19 are asymptomatic or have minimal symptoms, they can still spread the virus to vulnerable adults. The correct clinical diagnosis of mild cases of COVID-19 becomes often extremely difficult since the presenting symptoms could be similar with other common viral illnesses. Furthermore, each child may have several viral infections during the winter and in conjunction with insufficient supplies and the high cost of SARS-CoV-2 PCR-RT test in several countries makes the diagnosis of COVID-19 a very complicated one. We should also consider the whole cascade of consequence from children's lack of school attendance ranging from family's financial security to support the children's educational needs and the emotional well-being. The school absence could compromise the ability of parents to go to work due to possible lack of child care with major economic effects since in 73% of the families with school age children in the United States, both parents are employed (1). Similarly, the parental employment status in Australia has been reported to be about 69% (2), while in Europe the percentage of both parents having full time employment reaches 41% (3). Regarding alterations in education, COVID-19 pandemic has a catalytic impact on the educational system that had to adopt new learning modalities and move to distant learning. However, 31% of school age children around the globe do not have access to remote schooling (4). American Academy of Pediatrics strongly advocates the in person education for the present school year stressing the facts that staying away from school for a long time can result in social isolation and complicates the recognition and management of learning deficits (5). Even if blended educational curriculums have been implemented by several institutes (6), it seems that in person schooling has major advantages especially for young children. The goal is the children to attend school regularly and to minimize as much as possible the disruptive quarantine period with the major concern of the students' protection from COVID-19.

As the flu season is approaching in Northern hemisphere and the schools are reopened, the government officials are taking actions for managing the childhood minor respiratory infections in COVID-19 era. Several policies have been established in different countries with the goal to minimize the risk of transmission of SARS-CoV-2 (7–9). In addition, clinical algorithms treated children suspected with COVID-19 have been designed (10). However, the question remains as to when a child with symptoms of a viral illness can safely return to school reducing the unnecessary home stay. Trying to answer that question, we develop a return-to-school criteria for children with fever and/or cough combining several clinical and epidemiological parameters and taking into consideration the limited supply and the expense of SARS-CoV-2 PCR-RT test (Figure 1).

**Figure 1:**
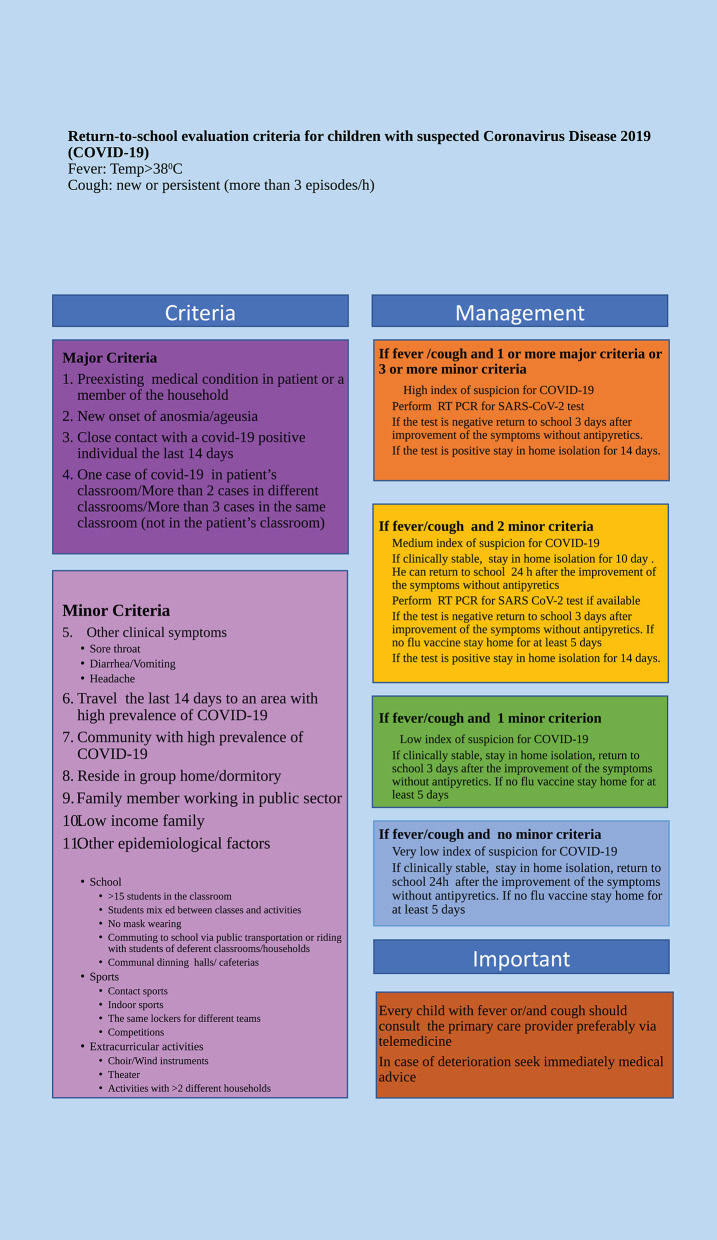
Return-to-school evaluation criteria for non-hospitalized children with suspected Coronavirus Disease 2019.

The goal of this algorithm is not only to make a safe decision to return-to-school but also to reduce the school absence as much as possible. Among the major criteria are the close, recent contact with a person with COVID-19, a vulnerable child or a family member and a new onset of anosmia and/or ageusia, characteristic symptoms for COVID-19 (11). One major criterion is enough to place the child in high risk and to require a SARS-CoV-2 RT-PCR test. The children can return to school if the test is negative, they are afebrile without antipyretics, and the other symptoms are improved for at least 3 days. Considering the possibility of a SARS-CoV-2 RT-PCR false negative test result, a 3-day interval with significant improvement of the symptoms without antipyretics was advised prior to return to school. The minor criteria consist of a combination of clinical and epidemiological factors. We select the most common epidemiological circumstances that place the child in very close proximity with other classmates during different activities at school, extra curriculum, sports, school dinning, and school transit. The community spread of the disease and living in close community have also been considered. A limit of 15 students in the classroom was selected based on the experience at Denmark and Norway. They have shown no increase in the rate of COVID-19 by decreasing the class size to <15 students (12). Another study contacting in Tokyo metropolitan area revealed that the class reduction and the social distancing of at least 1.5 m reduces the school closure due to flu pandemic by 90% (13). Centers of Disease Control and Prevention (CDC) and World Health Organization have published recommendations for schools, school dinning, school transit, and sports (7, 8). Those are based on maintaining physical distance among students during their activities. Several of those guidelines have been incorporated into our proposed stratification criteria. One of the minor criteria is the use of public transportation for school transit. A study in the Zhejiang province, China has been shown that riding a bus makes 42.2 (95% CI, 2.6–679.3) times more likely to develop COVID-19 if a fellow passenger is positive to SARS-CoV-2 (14). An additional criterion we include in the present algorithm is the use of face masks in the classroom. Many health authorities recommend the proper use of face masks by students and teachers (5, 8). Eikenberry et al. have evaluated the effectiveness of mask wearing in the community by developing model simulations. Those results indicate that the use of even moderately (50%) effective masks could prevent, on the range of 17–45%, the projected deaths over a 3 month period in New York City (15). In addition, an epidemiologic analysis have shown that the viral transmission from pre-symptomatic patients was significantly lower in mask-wearing persons compare to unmasked ones (19.0% vs. 8.1%, *p* < 0.001) (16). A family member working in the public sector has been also added as a minor criterion due to facilitation of community transmission of SARS-CoV-2 in that case. We have also included the family with low income status as it has been shown that the people living in poorer and more diverse areas have a high incidence of COVID-19 (17). The children with three or more minor criteria are considering as high risk and SARS-CoV-2 PCR-RT test is required. The cases with two minor criteria are assigned to medium risk and we propose to stay in home isolation for 10 days. SARS-CoV-2 PCR-RT test is advised if available. The children with none or one minor criterion are appointed to very low and low risk and they return to school when they are afebrile without antipyretics and the symptoms are improved for 24 h and 3 days, respectively. In the setting of no flu vaccine, the child should stay home for at least 5 days to minimize the risk of flu transmission and to avoid the co-infection between flu and COVID-19 (18, 19). Influenza immunization is strongly recommended during the present winter by the CDC (20). We would like to emphasize that the caregivers should consult their primary care physician regularly, during the home isolation period preferably via telemedicine and if the patient deteriorates they should ask immediately medical advice (10).

These stratification criteria, combining clinical and epidemiological factors, could be a useful tool for the primary care physicians to evaluate a child with fever and/or cough and make a safe return-to-school decision minimizing the unnecessary home stay. It is important, this proposed algorithm, to be revised as new data becomes available.

## Author Contributions

VV and GF contributed equally to the design, analysis, and writing of the manuscript.

## Conflict of Interest

The authors declare that the research was conducted in the absence of any commercial or financial relationships that could be construed as a potential conflict of interest.
